# Reconsidering Claims of Equivalence Between Oral and Intravenous Antibiotic Therapy for Infective Endocarditis: A Critical Appraisal of the Evidence

**DOI:** 10.1093/ofid/ofag694

**Published:** 2026-10-26

**Authors:** Dawd S Siraj, Fauzia Hollnagel

**Affiliations:** Division of Infectious Diseases, University of Wisconsin, Madison, Madison, Wisconsin, USA; Office of Biostatistics/Epidemiology, Department of Medicine, University of Wisconsin, Madison, Wisconsin, USA

**Keywords:** infective endocarditis, intravenous antibiotic therapy, non-inferiority trials, oral step-down therapy, *Staphylococcus aureus* bacteremia

In recent years, increasing attention has been directed toward oral antibiotic strategies for the treatment of high-consequence bacterial infections, particularly infective endocarditis (IE). Several observational studies, clinical trials, reviews, and commentaries have challenged traditional treatment paradigms and highlighted circumstances in which carefully selected patients may successfully complete therapy with oral agents. These studies have contributed importantly to the evolving discussion surrounding oral antibiotic therapy and have prompted renewed examination of longstanding treatment practices [1–4].

Although support for oral step-down therapy in highly selected, clinically stable patients has grown, questions remain regarding the extent to which the existing evidence supports broader claims of equivalence, non-inferiority, or earlier transition from intravenous (IV) to oral treatment. To better understand these issues, we reviewed the primary studies most frequently cited in support of oral antibiotic therapy for infective endocarditis and related high-consequence infections [5–12].

Our appraisal suggests that while these studies provide valuable clinical insights and have helped identify patient populations in whom oral completion therapy may be feasible, important methodological and interpretive limitations remain. These include heterogeneity in study design, patient selection, microbiology, therapeutic regimens, and study objectives. In addition, several frequently cited studies were not specifically designed or adequately powered to establish non-inferiority between oral and IV treatment strategies [11–13].

Given the substantial influence these publications have had on contemporary clinical discussions, we present a focused appraisal of eight influential studies that have shaped the evidence base for oral antibiotic therapy in infective endocarditis. Many of these studies have subsequently been cited in review articles, meta-analyses, and opinion papers as evidence supporting equivalence between oral and intravenous therapy. Our objective is not to challenge the potential role of oral therapy in carefully selected patients, but rather to examine the strengths and limitations of the existing literature and to highlight areas where additional rigorous investigation is needed before broad clinical generalizations can be made.

**Tissot-Dupont H. et al. High-Dose Trimethoprim-Sulfamethoxazole and Clindamycin for Staphylococcus aureus Endocarditis. Clin Infect Dis. 2017** [5].

In this single-center before–after open study, investigators compared outcomes of patients with *Staphylococcus aureus* infective endocarditis (SAIE) treated during two distinct periods: 2001–2011 as the control era and 2012–2016 as the intervention era. The study arose from a major institutional change in practice, replacing conventional 6-week intravenous therapy with a regimen consisting of seven days of intravenous trimethoprim-sulfamethoxazole (TMP-SMX) and clindamycin followed by oral TMP-SMX. Notably, the intervention strategy was associated with lower overall, in-hospital, and 30-day mortality, making this an important and provocative study that helped stimulate further interest in oral antibiotic strategies for infective endocarditis.

However, the study was not designed as a direct comparison of intravenous versus oral therapy. Rather, it compared two substantially different treatment approaches that differed in antimicrobial agents, route of administration, duration of therapy, and use of adjunctive treatments. Several methodological considerations should therefore temper interpretation of the findings:

1. Lack of a head-to-head comparison of equivalent regimens: The intervention differed from the control group in multiple respects beyond route of administration, making it difficult to attribute observed differences specifically to oral therapy.
2. Mandatory intravenous lead-in therapy: All patients in the intervention group received at least seven days of intravenous therapy, and oral treatment was initiated only after bacteremia clearance had been confirmed.
3. Use of additional parenteral agents: Approximately 28%–30% of patients in the intervention arm required adjunctive rifampin and gentamicin, further complicating attribution of outcomes to oral therapy alone.
4. Treatment crossover and discontinuation: Nearly 20% of patients assigned to the intervention strategy did not complete the planned regimen, requiring careful interpretation of the reported outcomes.

Although the intervention group demonstrated lower mortality and relapse rates, the study's design does not allow these benefits to be attributed specifically to the oral component of therapy. Nevertheless, the findings remain clinically important and helped generate interest in alternative treatment approaches for SAIE. In our view, however, this study should be regarded as hypothesis-generating rather than definitive evidence of non-inferiority between oral and intravenous therapy, and its results should be interpreted within the context of the methodological limitations described above.

**Mzabi A. et al. Switch to Oral Antibiotics in the Treatment of Infective Endocarditis. Clin Infect Dis. 2017** [6].

In this retrospective single-center study, investigators evaluated outcomes of patients with infective endocarditis (IE) managed under a locally developed protocol in which a multidisciplinary team determined eligibility for transition from intravenous to oral antibiotics. Transition occurred after a median of 21 days (range, 0–70 days) of intravenous therapy, following assessment of clinical response, microbiology, and disease severity. Importantly, this study addressed a clinically relevant question and provided valuable real-world experience regarding the feasibility of oral step-down therapy in carefully selected patients.

The authors appropriately acknowledged important baseline differences between groups and attempted to account for these imbalances through multivariable analysis. Patients transitioned to oral therapy had fewer comorbidities, lower severity at presentation, and were less frequently infected with *Staphylococcus aureus*, all characteristics generally associated with more favorable outcomes. After adjustment, the authors reported that oral transition was not associated with increased mortality.

Several methodological considerations, however, should temper interpretation of these findings:

1. Prolonged intravenous lead-in therapy: Patients received a median of 21 days of intravenous antibiotics before transition, representing a substantial portion of the standard treatment course for IE. As a result, the study provides limited insight into whether earlier oral transition would yield similar outcomes.
2. Retrospective, non-randomized treatment assignment: Selection for oral therapy was based on clinical judgment and patient characteristics, creating the potential for confounding by indication. Although multivariable adjustment was performed, residual confounding remains difficult to exclude.
3. Treatment decisions were closely linked to prognosis-related factors: Disease severity, comorbidities, microbiology, and timing of transition varied substantially between groups. While statistical adjustment can mitigate some of these differences, it cannot fully account for unmeasured factors that may have influenced both treatment selection and outcomes.

Taken together, this study provides encouraging evidence that oral step-down therapy may be feasible in carefully selected, clinically stable patients, particularly those with predominantly non-*S. aureus* IE who have already completed an extended course of intravenous therapy. However, given the observational design and the highly selected patient population, the findings should be viewed as supportive and hypothesis-generating rather than definitive evidence of non-inferiority or equivalence to standard intravenous therapy across the broader population of patients with infective endocarditis.

**Demonchy et al. Audit of Antibiotic Therapy Used in 66 Cases of Infective Endocarditis. Clin Microbiol Infect. 2018** [7].

This retrospective audit of 66 patients with infective endocarditis (IE) was primarily designed to evaluate the appropriateness of antibiotic prescribing rather than the comparative effectiveness of intravenous and oral treatment strategies. Nevertheless, the authors reported that 29% (19/66) of patients were transitioned to oral antibiotics and observed no significant difference in mortality between patients who underwent oral transition and those who remained on intravenous therapy. Although not designed to answer the question of oral versus intravenous therapy, the study contributed valuable real-world observations regarding treatment practices and helped generate interest in alternative approaches to IE management.

Several methodological considerations, however, should temper interpretation of these findings:

1. Small observational study population: The study was neither designed nor powered to evaluate equivalence or non-inferiority between oral and intravenous therapy. Consequently, the absence of a statistically significant difference should not be interpreted as evidence that the two approaches are comparable.
2. Substantial intravenous lead-in therapy: Patients who transitioned to oral treatment had already received an average of 18 ± 9 days of intravenous antibiotics, representing a considerable portion of the standard treatment course for IE. As a result, the study provides limited information regarding the effectiveness of oral therapy itself.
3. Limited characterization of patient selection: The study does not provide detailed information regarding baseline comorbidities, disease severity, or the clinical rationale for selecting patients for oral transition. Without these data, it is difficult to determine whether patients receiving oral therapy differed systematically from those who remained on intravenous treatment.

Taken together, this study provides a useful description of clinical practice and suggests that oral transition may be feasible in selected patients who have already completed a substantial course of intravenous therapy. However, given its observational design, small sample size, and limited information regarding patient selection, it should be viewed as hypothesis-generating rather than definitive evidence regarding the equivalence or non-inferiority of oral and intravenous treatment strategies for infective endocarditis.

**Stamboulian D. et al. Antibiotic Management of Outpatients with Endocarditis Due to Penicillin-Susceptible Streptococci** [8].

This prospective study evaluated the efficacy of ceftriaxone-based treatment for infective endocarditis caused by penicillin-susceptible streptococci, comparing two regimens: four weeks of intravenous (IV) ceftriaxone versus two weeks of IV ceftriaxone followed by two weeks of oral amoxicillin. All 30 enrolled patients had endocarditis caused by highly penicillin-susceptible streptococcal isolates. Although modest in size, the study was among the earlier prospective efforts to explore whether selected patients with infective endocarditis could safely complete therapy with an oral regimen.

Several important limitations, however, should temper interpretation of the findings:

1. Small sample size and narrow microbiologic scope. The study enrolled only 30 patients (15 per treatment arm), all infected with a single pathogen characterized by predictable susceptibility to β-lactam antibiotics. While outcomes appeared similar between groups, the study was not adequately powered to support a meaningful non-inferiority comparison, and the highly selected patient population limits broader applicability of the findings.
2. Delayed transition to oral therapy. Patients in the oral arm completed two full weeks of intravenous ceftriaxone before transitioning to oral amoxicillin. Consequently, the study evaluates the feasibility of completing treatment with oral therapy after a substantial IV lead-in rather than providing a direct comparison between predominantly IV and oral treatment strategies.
3. Exclusion of higher-risk patient populations. Patients with significant valvular dysfunction, conduction abnormalities, recurrent embolic events, prosthetic valve endocarditis, and heart failure were excluded. These restrictions substantially narrow the clinical spectrum represented in the study and limit applicability to more complex forms of infective endocarditis.

Importantly, the investigators interpreted their findings cautiously and appropriately limited their conclusions to a highly selected subgroup of patients with uncomplicated streptococcal endocarditis. The study provides valuable preliminary evidence that oral completion therapy may be feasible in carefully chosen patients after an initial course of intravenous treatment. However, given its small sample size, restricted patient population, and substantial IV lead-in period, it should be viewed as supportive and hypothesis-generating rather than definitive evidence of non-inferiority between oral and intravenous treatment strategies for infective endocarditis more broadly.

**Heldman AW et al. Oral Antibiotic Treatment of Right-Sided Staphylococcal Endocarditis in Injection Drug Users: A Prospective, Randomized Comparison with Parenteral Therapy** [9].

This prospective randomized study evaluated a highly selected population of injection drug users with right-sided *Staphylococcus aureus* infective endocarditis (IE). Investigators compared an oral regimen of ciprofloxacin plus rifampin (19 patients) with standard intravenous therapy consisting of oxacillin or vancomycin (25 patients), both administered with gentamicin. The authors reported similar cure rates between groups and concluded that oral therapy represented a potential alternative in this specific clinical setting. As one of the earliest randomized studies examining an entirely oral treatment strategy for IE, this trial made an important contribution to the discussion regarding oral antibiotic therapy.

Several limitations, however, should be considered when interpreting the results:

1. Small sample size and restricted clinical scope.
  The study enrolled only 44 patients and was limited exclusively to injection drug users with uncomplicated right-sided *S. aureus* IE. Consequently, the findings cannot be readily extrapolated to patients with left-sided disease, complicated endocarditis, prosthetic valve infection, or non–injection drug-using populations.
2. Failure to achieve the planned sample size.
  Based on the investigators’ own power calculations, 84 participants were required to detect a clinically meaningful difference in cure rates. However, enrollment ended after the predetermined two-year recruitment period with only 44 patients enrolled, approximately half of the intended sample size. This shortfall substantially reduced the study's statistical power and limits confidence in conclusions regarding comparative efficacy.
3. Imprecise treatment effect estimates.
  Although outcomes appeared similar between groups, both the primary and secondary analyses were associated with wide confidence intervals. These broad intervals reflect the limited sample size and indicate considerable uncertainty regarding the true magnitude of any difference between treatment strategies.

In summary, this study represents an important early randomized evaluation of oral therapy for infective endocarditis and suggests that selected patients with uncomplicated right-sided *S. aureus* IE may be successfully treated with an oral regimen. However, the small sample size, incomplete enrollment, and resulting imprecision of the treatment effect estimates limit the strength of the conclusions. The findings are best viewed as supportive and hypothesis-generating rather than definitive evidence of non-inferiority, particularly with respect to broader populations of patients with staphylococcal endocarditis.

**Kaasch AJ et al**. **Efficacy and safety of an early oral switch in low-risk *Staphylococcus aureus* Bloodstream Infection (SABATO). Lancet Infectious Diseases. 2024;24:523–534** [10].

The SABATO trial was a multicenter, randomized, non-inferiority study evaluating early oral switch therapy in patients with low-risk *Staphylococcus aureus* bacteremia. As one of the largest contemporary randomized trials addressing oral antibiotic therapy for a high-consequence bacterial infection, the study represents an important contribution to the field and reflects the growing interest in developing alternatives to prolonged intravenous treatment.

Several aspects of the study, however, warrant careful consideration when interpreting the findings:

1. Modification of sample size and non-inferiority margin.
  Due to slower-than-anticipated recruitment, the study protocol was amended during the trial, reducing the target sample size from 430 to 213 participants. This adjustment was achieved largely by expanding the non-inferiority margin from 5% to 10%. While such modifications are methodologically permissible, they materially influence the clinical interpretation of non-inferiority and may increase the likelihood of concluding non-inferiority despite potentially important differences in treatment efficacy.
2. Highly selected study population.
  Of more than 5000 patients screened, only 213 were ultimately randomized. Participants were carefully selected and limited to patients with uncomplicated bacteremia and without many of the comorbidities and complications frequently encountered in clinical practice. As a result, the findings may not be readily generalizable to the broader population of patients with *S. aureus* bacteremia.
3. Prior intravenous therapy before oral transition.
  Patients received 5–7 days of intravenous antibiotics before switching to oral therapy. Consequently, the trial evaluates a partial oral treatment strategy following an initial course of effective IV therapy rather than a true early oral treatment approach.

In summary, the SABATO trial provides important evidence that oral step-down therapy may be feasible in carefully selected patients with uncomplicated *S. aureus* bacteremia following initial clinical stabilization and intravenous treatment. However, the protocol modifications, highly selected patient population, and requirement for initial IV therapy suggest caution when extending these findings beyond the population studied. As with other studies in this area, the results are best interpreted within the context of the trial's design and patient selection criteria.

**Iversen K. et al. Partial Oral versus Intravenous Antibiotic Treatment of Endocarditis (POET Trial). N Engl J Med. 2019;380:415–424** [11].

The POET trial was a multicenter, randomized non-inferiority study comparing partial oral therapy with continued intravenous (IV) therapy in clinically stable patients with left-sided infective endocarditis (IE). The primary composite outcome included all-cause mortality, unplanned cardiac surgery, embolic events, or relapse of bacteremia. Importantly, patients were eligible for randomization only after demonstrating clinical stability, clearance of bacteremia, favorable response to initial IV therapy, and absence of intracardiac abscess. As the largest and most rigorous trial evaluating oral step-down therapy for IE, POET represents a landmark contribution to the field and has appropriately influenced subsequent discussions regarding oral antibiotic management.

Several aspects of the study, however, warrant careful consideration when interpreting its findings:

1. Choice of non-inferiority margin.
  The investigators selected an absolute non-inferiority margin of 10 percentage points despite an anticipated event rate of approximately 5%–13%. While this approach is statistically acceptable, it permits a potentially substantial reduction in efficacy while still meeting criteria for non-inferiority. Whether such a margin is clinically acceptable for a high-consequence infection such as left-sided IE remains open to debate.
2. Timing of randomization and extent of IV lead-in therapy.
  Patients were randomized only after a median of 17 days (IQR, 13–24) of IV therapy. Consequently, much of the intended treatment course had already been completed intravenously before oral therapy was initiated. The trial therefore primarily examines the safety of completing therapy orally after a prolonged period of clinical stabilization rather than evaluating an early oral treatment strategy.
3. Crossovers and interpretation of ITT analyses.
  Several patients assigned to oral therapy ultimately returned to IV treatment but remained in the oral group under the intention-to-treat analysis. While this approach is methodologically appropriate and standard for randomized trials, such crossovers can bias non-inferiority studies toward finding equivalence and should be considered when interpreting the results.
4. Highly selected study population.
  Only a minority of screened patients ultimately underwent randomization. Patients with persistent bacteremia, abscess formation, hemodynamic instability, and other common complications of IE were excluded. As a result, the findings apply primarily to a carefully selected population that had already demonstrated a favorable response to treatment.

In summary, the POET trial provides important evidence that completion of therapy with oral antibiotics may be a reasonable option in carefully selected patients with left-sided infective endocarditis who have achieved sustained clinical stability after more than two weeks of IV therapy. At the same time, the stringent eligibility criteria, prolonged IV lead-in period, and inherent challenges of non-inferiority trial design suggest caution when extending these findings beyond the population studied. Notably, the investigators themselves acknowledged these limitations and appropriately avoided unrestricted extrapolation of their results to the broader population of patients with infective endocarditis.

**Juskowich JJ et al. Using the Comparing Oral versus Parenteral Antimicrobial Therapy (COPAT) Clinical Trial to Influence Institutional Practice Transformation Towards Earlier Transition to Oral Antibiotics. Clin Infect Dis. 2025** [12].

In this study, the investigators compared adverse events and clinical outcomes among patients treated with oral versus intravenous (IV) antibiotic therapy. The study addresses an important and clinically relevant question, particularly as interest in earlier oral transition continues to grow. However, several aspects of the study design warrant careful consideration when interpreting the findings.

1. Study powered for safety rather than efficacy.
  The primary sample size calculation was based on demonstrating differences in adverse events rather than treatment efficacy. As a result, the study was not adequately powered to establish non-inferiority with respect to infection cure or control, the outcome most likely to influence clinical decision-making in serious infections.
2. Early trial termination.
  Enrollment was stopped after approximately two-thirds of the planned sample size had been accrued because of a reported safety benefit. While this finding is noteworthy, early termination further reduced the number of participants available for meaningful comparisons of clinical efficacy.
3. Interpretation of efficacy outcomes.
  Although efficacy outcomes were reported, the limited sample size means that an absence of statistically significant differences should be interpreted cautiously. Failure to detect a difference in an underpowered study does not necessarily establish equivalence or non-inferiority between treatment strategies.

In summary, this study contributes important information regarding the safety and feasibility of oral antibiotic therapy and adds to the ongoing discussion surrounding oral treatment strategies. However, because the trial was designed and powered primarily around safety rather than efficacy outcomes, the findings are best viewed as supportive and hypothesis-generating rather than definitive evidence of non-inferiority for early transition to oral therapy.

## CONCLUSION

In summary, our review suggests that an oral step-down approach for the treatment of infective endocarditis (IE) may be a reasonable consideration in carefully selected patients, particularly those who have achieved clearance of bacteremia, demonstrated sustained clinical stability, and are infected with organisms for which highly bioavailable oral agents are available. Importantly, in the studies discussed, transition to oral therapy generally occurred only after a substantial period of initial intravenous (IV) treatment.

Reassuringly, a recent Emerging Infections Network survey of US infectious diseases physicians found that partial oral therapy for infective endocarditis remains relatively uncommon in routine clinical practice. This continued caution among clinicians underscores the importance of ongoing critical appraisal and open scientific discussion as the role of oral therapy in infective endocarditis continues to evolve. While the authors suggested that future guideline updates may facilitate broader adoption of this approach, we believe that any expansion in use should remain closely aligned with the strength and scope of the available evidence.

The existing literature has made important contributions to our understanding of oral antibiotic strategies and has helped identify patient populations in whom oral completion therapy may be feasible. However, the evidence supporting broader claims of equivalence, non-inferiority, or earlier transition to oral therapy remains limited. The studies most frequently cited in support of these approaches are characterized by substantial heterogeneity in design, patient selection, microbiology, treatment regimens, and study objectives. Moreover, many were not specifically designed or adequately powered to establish non-inferiority between oral and IV treatment strategies [13, 14].

It is also important to recognize that the fact that historical standards of care were not developed under modern clinical trial methodology does not diminish their established clinical effectiveness. Non-inferiority trial design was developed precisely to evaluate alternative treatment strategies while preserving confidence that meaningful efficacy is not sacrificed in pursuit of advantages such as reduced cost, greater convenience, or improved tolerability. For this reason, observational studies, retrospective analyses, and low-quality meta-analyses, while valuable for hypothesis generation, cannot fully substitute for the rigor of adequately designed non-inferiority trials when considering changes to established standards of care for high-consequence infections [15, 16].

Taken together, these considerations suggest caution in generalizing equivalence between oral and IV therapy for high-consequence infections like infective endocarditis [17]. Rather than diminishing the value of the existing literature, these limitations highlight the need for additional rigorously designed, adequately powered, multicenter studies to better define which patients may safely benefit from oral treatment strategies and to establish the boundaries within which those findings can be confidently applied [18, 19]. We also believe that investigators, peer reviewers, journals, and research sponsors all play an important role in ensuring that non-inferiority studies are interpreted within the context of their methodological assumptions, study populations, and intended scope of inference [20–22]. Given the potential for such studies to influence clinical practice, careful attention to trial design, statistical methodology, analytic approaches, and generalizability remains essential. Continued emphasis on these principles will help ensure that evolving treatment strategies are adopted on the basis of robust evidence and that conclusions drawn from individual studies are applied appropriately to patient care. Until such data are available, a measured and individualized approach to oral step-down therapy remains both reasonable and consistent with the principles underlying evidence-based adoption of new treatment strategies.
